# Development of unigene-derived SSR markers from RNA-seq data of *Uraria lagopodioides* (Fabaceae) and their application in the genus *Uraria* Desv. (Fabaceae)

**DOI:** 10.1186/s12870-023-04086-1

**Published:** 2023-02-10

**Authors:** Chaoyu Liu, Maomao Zhang, Xueli Zhao

**Affiliations:** 1 College of Forestry, Southwest Forestry University, Kunming, 650224 China; 2 Key Laboratory of National Forestry and Grassland Administration on Biodiversity Conservation in Southwest China, Southwest Forestry University, Kunming, 650224 China

**Keywords:** *Uraria*, Transcriptome sequencing, Unigene-derived SSR, Interspecific transferability, Genetic diversity

## Abstract

**Background:**

*Uraria* Desv. belongs to the tribe Desmodieae (Fabaceae), a group of legume plants, some of which have medicinal properties. However, due to a lack of genomic information, the interspecific relationships, genetic diversity, population genetics, and identification of functional genes within *Uraria* species are still unclear.

**Results:**

Using RNA-Seq, a total of 66,026 *Uraria lagopodioides* unigenes with a total sequence content of 52,171,904 bp were obtained via de novo assembly and annotated using GO, KEGG, and KOG databases. 17,740 SSRs were identified from a set of 66,026 unigenes. Cross-species amplification showed that 54 out of 150 potential unigene-derived SSRs were transferable in *Uraria*, of which 19 polymorphic SSRs were developed. Cluster analysis based on polymorphisms successfully distinguished seven *Uraria* species and revealed their interspecific relationships. Seventeen samples of seven *Uraria* species were clustered into two monophyletic clades, and phylogenetic relationships of *Uraria* species based on unigene-derived SSRs were consistent with classifications based on morphological characteristics.

**Conclusions:**

Unigenes annotated in the present study will provide new insights into the functional genomics of *Uraria* species. Meanwhile, the unigene-derived SSR markers developed here will be invaluable for assessing the genetic diversity and evolutionary history of *Uraria* and relatives.

**Supplementary Information:**

The online version contains supplementary material available at 10.1186/s12870-023-04086-1.

## Background

*Uraria* is a genus of legumes that contains ca. 20 species that are mainly distributed in tropical and subtropical Asia, Australia, and Africa [1–3]. Several species of *Uraria* (e.g., *U. lagopodioides*, *U. crinita* and *U. picta*) are used for medicinal purposes. The flavonoids, triterpenes, megastigmanes, nucleoside compounds, and 3-hydroxy-7’,4’-dimethoxyflavone they produce display a wide range of medicinal properties used in the treatment of asthma, dysentery, ulcers, and malaria-induced fever [4–7].

Previous studies of *Uraria* have mainly focused on morphology, geographical distribution, and palynology, with limited phylogenetic analyses of *Uraria* within the tribe Desmodieae. [1–3, 8–17]. Jabbour et al*.* conducted molecular phylogenetic and historical biogeographic analyses of Desmodieae genera endemic to New Caledonia using both nuclear (ITS1) and chloroplast (*rbc*L, *psb*A-*trn*H) fragments, though only three *Uraria* species were included [18]. Ohashi et al*.* provided a new classification of Desmodieae using a single nuclear (ITS) and nine chloroplast (5’*trn*K intron, *ndh*J-*trn*L-*trn*F, *trn*T-*trn*L, *trn*G-*trn*S, *trn*Q-*rps*16, *trn*L-*rp*l32, *rpl*16 intron, *trn*C-*rpo*B and *ndh*A intron) fragments, including sequences from six *Uraria* species. New taxonomic treatments were proposed based on phylogenetic analyses with morphological and palynological characters. *Desmodium oblongum* Wall. ex Benth. was transferred to *Uraria*, as a synonym of *Uraria oblonga* (Wall. ex Benth.) H. Ohashi & K. Ohashi [19]. However, the phylogenetic relationships and evolutionary history of *Uraria*, especially for phylogenetically related species*,* are still largely uncharacterized.

DNA-based molecular markers such as restriction length fragment polymorphisms (RFLPs), random amplified polymorphic DNAs (RAPDs), and simple sequence repeats (SSRs) have been employed effectively in numerous studies of genetic diversity [20–22], among which SSRs are popular for the differentiation of heterozygotes and homozygotes, reliable reproducibility, and cost-effectiveness. SSRs consist of tandem units of short nucleotide motifs of 1–6 bp in length [23–26]. SSRs can be developed from both non-expressed regions and expressed regions, referred to as genomic SSRs and genic SSRs, respectively [27–30]. Unigenes from the expressed regions are the longest transcripts in genes and have been widely used for SSR marker development. Compared to genomic SSRs, unigene-derived SSRs are more likely to be transferable and orthologous and have been widely used in phylogenetic and population genetic studies, especially for analyses of genetic diversity among phylogenetically related species [31, 32]. Next-generation sequencing, especially RNA-Seq using an Illumina platform, has been used as a rapid and cost-effective solution for identifying and developing SSR markers in non-model plants [33–35].

The objectives of this study were: (1) to enrich *Uraria* transcriptome data and better understand the functional significance of expressed genes, (2) to develop unigene-derived SSRs and examine both their cross-species transferability and levels of polymorphism, and (3) to reconstruct the genetic relationships of *Uraria* species.

## Results

### Illumina sequencing and de novo transcriptome assembly

A total of 8.23 Gb of clean data were obtained, and the Q20, Q30, and GC contents were 97.34%, 92.37%, and 43.98%, respectively. A total of 66,026 unigenes were assembled, of which there were 337,837 unigenes with a length of 200–500 bp, 12,769 with a length of 500–1000 bp, 9,297 with a length of 1–2 kb, and 6,123 with a length of more than 2 kb. The N50 of the unigenes was 1,850, indicating a high-quality assembly.

### Functional annotation of unigenes

To annotate *U. lagopodioides* unigenes, sequences from 66,026 unigenes were searched against different universal databases. 31,065 (47.04%) unigenes were aligned to sequences in the Nr database, 35,722 (54.10%) in the Nt database, 23,160 (35.07%) in the Swiss-Prot database, and 21,178 (32.07%) in the Pfam database. The annotation of 39,915 (60.45%) unigenes was achieved in at least one database.

**Fig. 1:**
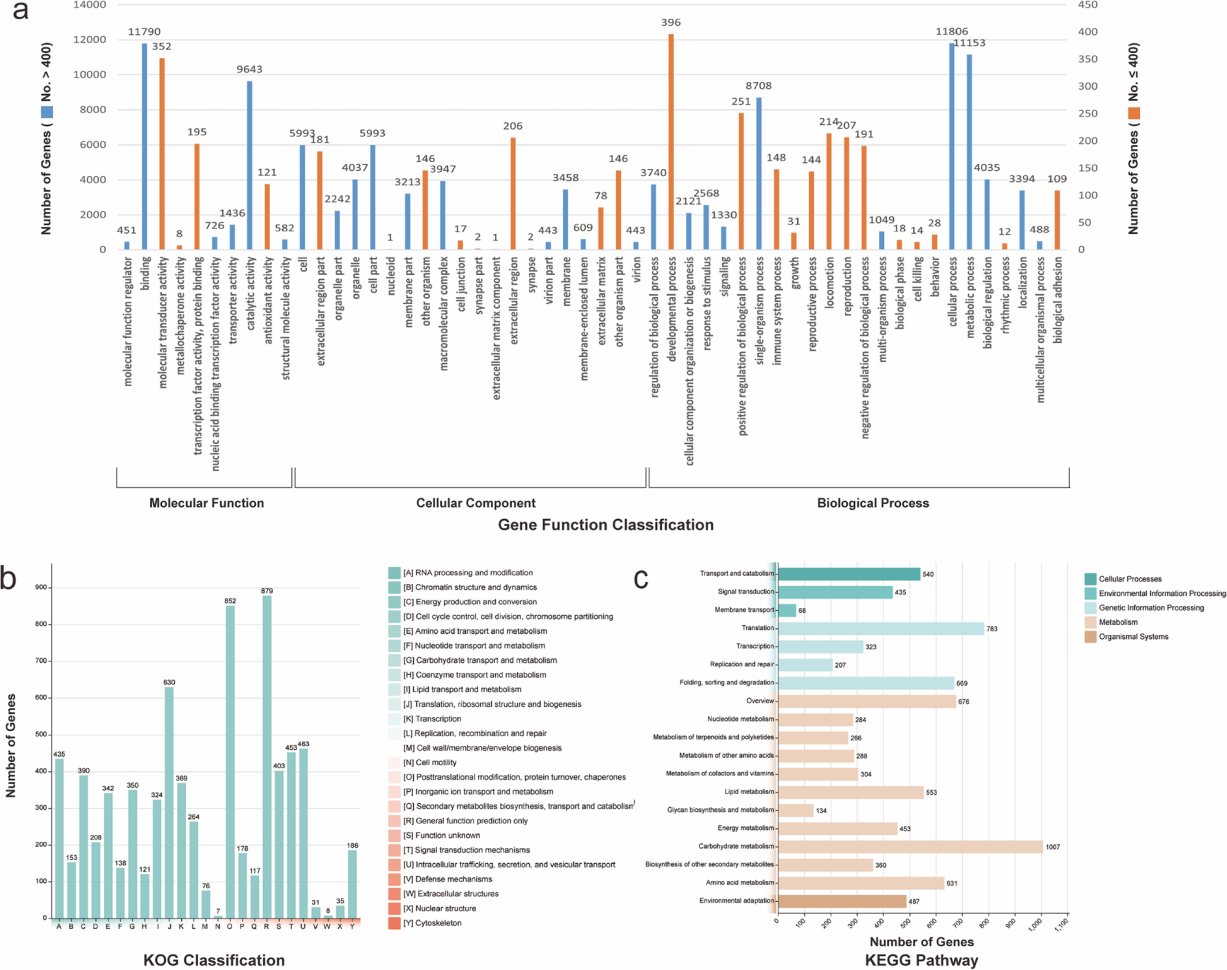
Gene annotations of *U. lagopodioides* unigenes based on GO, KOG, and KEGG databases. **a**. GO annotations of *U. lagopodioides* unigenes. **b**. KOG classifications of *U. lagopodioides* unigenes. **c**. KEGG classifications of *U. lagopodioides* unigenes

According to gene ontology (GO) analyses, 21,178 (32.08%) annotated unigenes could be assigned to three functional categories: biological processes, molecular functions, and cellular components (Fig. 1a). In “biological process”, the largest classes were “cellular process” (11,806, 17.88%), “metabolic process” (11,153, 16.89%), and “single organization process” (8,708, 13.19%). The cellular component category mainly consists of genes assigned to “cell” (5,993, 9.08%) and “cell part” (5,993, 9.08%) categories. The largest class identified in the molecular function category was “binding” (11,790, 17.86%). According to the KOG database, 6,614 unigenes (10.01%) were categorized into 25 functional groups (Fig. 1b), of which 879 were annotated as “general functional” genes, followed by “post-translational modification, protein turnover, chaperones” (852), and “translation, ribosomal structure, and biogenesis” (630). “Cell motility” (7) and “extracellular structures” (8) were the least frequently observed KOG classifications. According to the KEGG database, 10,956 unigenes were categorized into 19 biological pathways in five large groups (cellular processes, environmental information processing, genetic information processing, metabolism, and organismal systems) (Fig. 1c). Among them, the three most frequently observed functional pathways were “carbohydrate metabolism” (1,007), “translation” (783), and “overview” (676).

### Identification and characteristics of unigene-derived SSRs

A total of 17,740 potential unigene-derived SSRs were identified from the set of 66,026 unigenes (52,171,904 bp), with 2,952 unigenes containing more than one SSR locus. Of the 17,740 SSRs, 1,156 were presented in compound formation. These SSRs were further divided into six different types based on unit size, of which the mono-nucleotide repeats exhibited the highest frequency of occurrence (10,769, 60.70%), followed by tri-nucleotides (3,337, 18.81%), di-nucleotides (3,288, 18.53%), tetra-nucleotides (288, 1.62%), penta-nucleotides (36, 0.20%), and hexa-nucleotides (22, 0.12%) (Table 1). The most frequent mono-nucleotide repeats were A/T (10,635), accounting for 59.95% of the total SSRs. Of the tri-nucleotide repeats, AAG/CTT (799, 4.50%) was the most abundant motif, followed by AAT/ATT (662, 3.73%) and AAC/GTT (558, 3.15%). The most abundant di-nucleotide, tetra-nucleotide, and penta-nucleotide repeats were AG/CT (1,731, 9.76%), AAAT/ATTT (68, 0.38%), and AACAC/GTGTT (3, 0.02%), respectively. The number of repeats ranged from 5 to 36, with 10, 5, and 6 being the most frequent (Additional file 1: Table S1).

**Table 1:** Length distributions of the unigene-derived SSRs of *U. lagopodioides* based on the number of nucleotide repeat units

No. of repeats	Mono-	Di-	Tri-	Tetra-	Penta-	Hexa-	Total	Percentage (%)
5			1789	251	34	7	2081	11.73
6		1082	919	31	1	9	2042	11.51
7		595	588	5	1	4	1193	6.72
8		462	35			2	499	2.81
9		504	2	1			507	2.86
10	3234	480	4				3718	20.96
11	1776	158					1934	10.9
12	1146	5					1151	6.49
13	815	1					816	4.6
14	659						659	3.71
15	582						582	3.28
16	534						534	3.01
17	489						489	2.76
18	511						511	2.88
19	465						465	2.62
20	324						324	1.83
21	163						163	0.92
22	54						54	0.3
23	15						15	0.08
24	2						2	0.01
36		1					1	0.01
Total	10,769	3288	3337	288	36	22	17,740	
Percentage (%)	60.7	18.53	18.81	1.62	0.2	0.12		

**Fig. 2:**
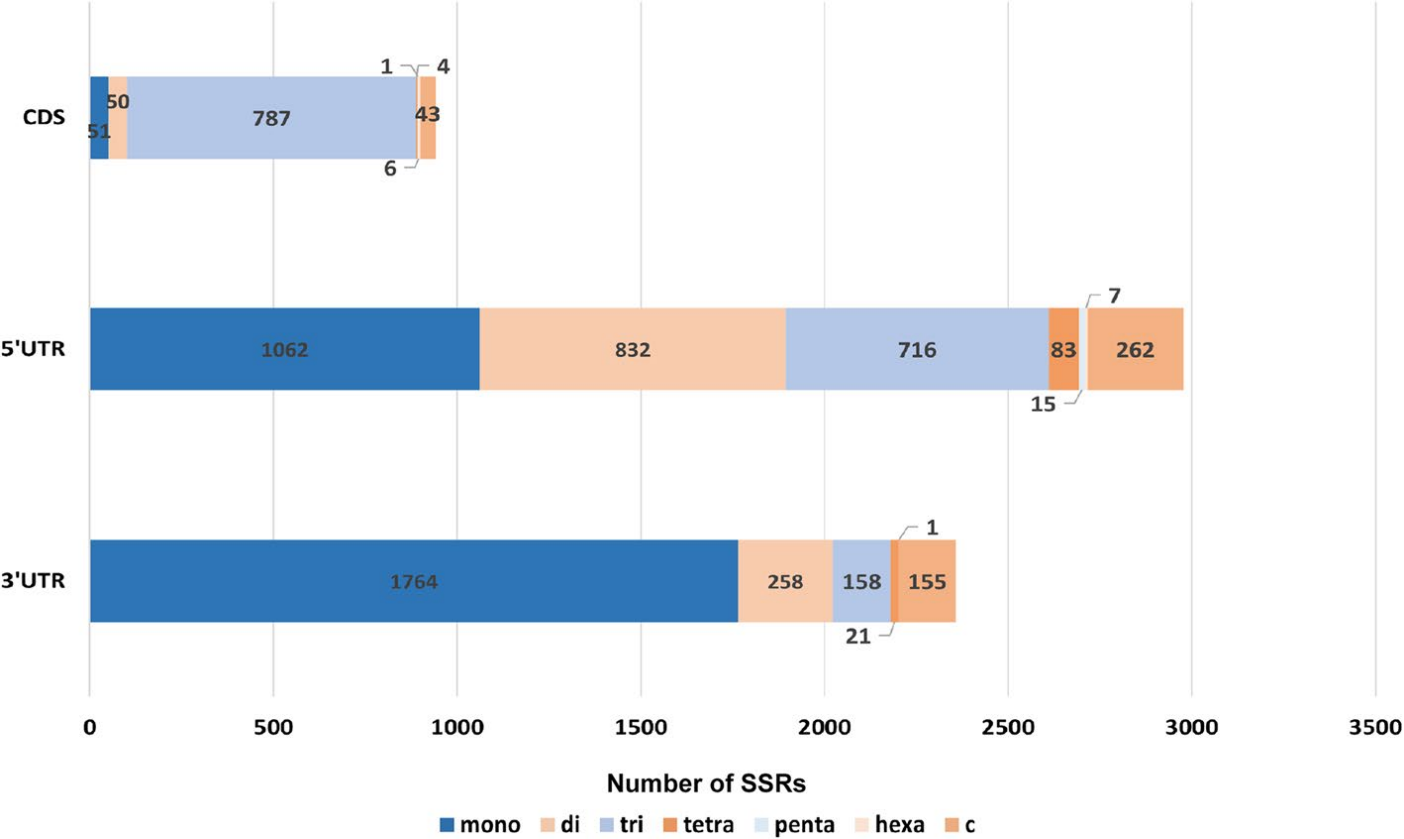
Frequency and distribution of SSRs in coding sequence (CDS) and untranslated region (UTRs) of *U. lagopodioides*

We analyzed the distribution of SSRs in the 3' UTR, 5' UTR and CDS regions of the genome (Fig. 2). There were 2,977, 2,357 and 942 SSRs distributed in 5'UTR, 3'UTR and CDS, respectively. The trinucleotide repeat sequence is the most abundant in the CDS region. The number of SSRs in the UTR region was significantly higher than that in the CDS region, and most SSRs were distributed in 5' UTR.

### Development of polymorphic unigene-derived SSR markers

To validate primers designed to detect unigene-derived SSRs, 150 potential unigene-derived SSRs were randomly selected and tested in *Uraria*. Fifty-four of these were successfully amplified and produced amplicons of expected size using genomic DNA as a template, while the remaining 96 failed to amplify despite trying a range of annealing temperatures (Additional file 2: Table S2). Using 17 individuals from seven *Uraria* species, 19 of 54 unigene-derived SSRs showed high levels of polymorphism and good transferability among different *Uraria* species (Table 2).

**Table 2:** Characteristics of 19 polymorphic unigene-derived SSRs

SSR locus	Forward primer (5'-3')	Reverse primer (5'-3')	Repeat motif	Ta (℃)	Observed product size (bp)	Na
P6	GACACGCTCATGTCGCATTC	GAGCATTTGAAGTCGGTGCG	(TG)8	55	194–227	8
P8	ACGGTCGATGAGTTTGTCGT	ACTGCTGTGCTGCTGTAGAA	(AG)8	55	231–297	2
P25	TGATGTCCTCCACATGTGCC	GCCACTCACCTAGAAGAGCC	(GTGA)7	55	268–281	5
P30	AGATTGCAGCAAGGGTCTCA	GGCACCTCCAATTCCTAGTCA	(TTCT)5	54	177–195	3
P32	CCAGCAGGTACATGTCTGGG	AGGTATTGTAGTCAAAGAACCAGT	(GT)9	53	168–177	3
P38	AGAGTTGCGACGCATAACCA	CCGTAAAGTCCGCGTGAAAC	(TA)8	55	225–265	3
P39	ACGCATATCGGGTCAGTAAACA	GTATTACAAATATTACATGCACACTGC	(GT)6(T)11	53	205–247	5
P40	TGGTTGACCGCAGCATAGTT	GAAGGTGCTTGTCCATGGGA	(TGG)6	55	458–498	7
P43	TGCTGCACCCTTCACATTCA	GGGTCGAGTTTGAGGAGTCG	(AC)8	55	224–255	4
P46	GGAGCAATGTCACCAAGGGA	CCATCTTAGAGCTGGCCACA	(GATG)5	55	355–370	1
P55	GCAGGTTTACGTCCAATGCT	ACAGTGTGCCCTCACGAAAT	(TATC)5	54	217–247	3
P59	ACCCTTCACAAACTCCCTCTC	AGGCTGAAAGAATGGCCTCC	(TTG)6	54	260–274	2
P71	CCACACCACAGCAGAACAGA	GTTCGGGCAAGGGAAGAGAA	(AC)6*(CT)9	55	154–225	10
P127	TCAACCACCGTAGCTGAACC	CGGATCTGAGAGCGGAAGAC	(GGA)6	55	231–277	5
P131	TGACAAGCCTTCCCGAACTC	CTCTAGGCACCTGCAAGAGA	(TTTA)5	54	243–251	2
P132	CGTTCGAGACCCAAGTCAGT	AGCATCACAGCCATCAACGA	(ATTA)5	55	237–286	2
P138	CAGCGTCCTCTGTGTTCTGT	AGTTCCGATTTCCGAAGCGA	(AT)9	55	217–285	7
P141	GAGGAGAAGGCGGTGATGAC	TAGAAGCAGCACCACCACTG	(TGG)7	55	275–296	3
P149	ACCGCAACTCATTACCTCCT	AGAATTCAGCACACGGAGCA	(TA)10	54	120–176	5
*GCACCTTACTCACTCTCTGTTACTCTTCACACA						

### Cluster analysis of *Uraria* based on unigene-derived SSRs

**Fig. 3:**
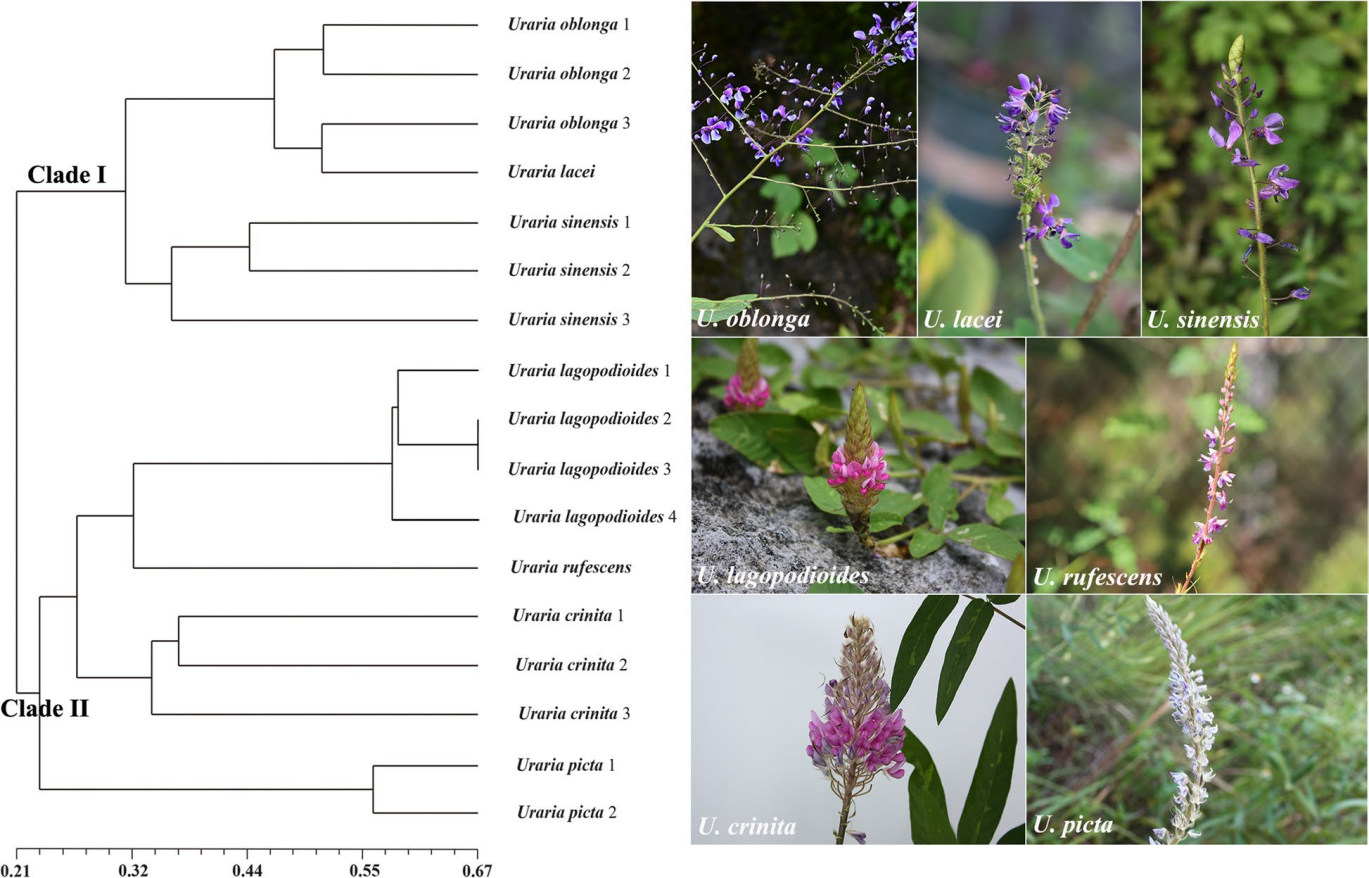
UPGMA cluster analysis of *Uraria* species

The *r*-value of matrix correlation was 0.847, and the value of the approximate mantel t-test was 9.869. The topology of the unweighted pair-group method analysis (UPMGA) tree based on genetic distance was used to show the relationships of *Uraria* species (Fig. 3). UPMGA cluster analysis revealed that 17 samples from seven *Uraria* species were clustered into two monophyletic clades. *Uraria oblonga*, *U. lacei*, and *U. sinensis* were clustered into Clade I. *U. lagopodioides*, *U. rufescens*, *U. crinita*, and *U. picta* were clustered into Clade II, indicating close genetic relationships.

## Discussion

RNA-Seq is a cost-efficient and powerful technology for generating high-coverage transcriptome data, and it has been increasingly used for detecting functional genes and identifying molecular markers in non-model plants such as *Panax vietnamensis* [36], *Acrocomia aculeata* [37], *Luculia yunnanensis* [38], *Paris polyphylla* [39], and *Bromus catharticus* [31]. However, no transcriptome sequencing of *Uraria* species has been reported thus far. In the present study, we reported the first transcriptome sequence data of *U. lagopodioides* using Illumina RNA-Seq technology. A total of 55,933,282 paired-end raw reads were generated, of which 54,843,810 were high-quality clean reads. 97.34% of reads had minimum quality scores of Q20, indicative of high-quality sequencing [28, 40, 41].

Previous studies have shown that unigenes longer than 500 bp are more amenable to annotation efforts, while reads with shorter lengths are more difficult to annotate and categorize [42–45]. In the present study, a total of 66,026 unigenes were assembled from the *U. lagopodioides* transcriptome with an average length of 1,041 bp and N50 length of 1,850 bp, which was longer than those reported in the studies of *Panax vietnamensis* (598.32 bp and 1,268 bp, respectively) [36], *Brassica napus* (834 bp and 1,245 bp) [46], *Parrotia subaequalis* (890 bp and 1,591 bp) [47], and *Vigna aconitifolia* (937.78 bp and 1,227 bp) [48], but shorter than *Phoebe bournei* (1,019 bp and 2,016 bp) [49] and *Lathyrus sativus* (1,250 bp and 1,781 bp) [50]. The unigenes generated in this study will be valuable for characterizing molecular mechanisms and exploring novel functional genes in *Uraria* and related taxa. To obtain comprehensive gene function categories of *U. lagopodioides*, we performed gene function annotations using the public databases KOG [51], GO [52], and KEGG [53]. In sum, 4,095 of 66,026 unigenes were functionally annotated in all three databases, and 39,915 were functionally annotated in at least one database. The low percentage of annotated unigenes may be a consequence of the relative dearth of related species in these databases or a relatively large proportion of non-coding regions in the *U. lagopodioides* transcriptome sequence [38, 40, 54, 55].

As a result of gene function annotation, 21,178 unigenes (32.07%) were classified into GO categories. The largest GO category was “cellular process”, followed by “binding”. A total of 10,956 unigenes (16.59%) were annotated using the KEGG database, with the largest group of genes categorized as “carbohydrate metabolism”, followed by “translation”. According to the KEGG database, many unigenes were classified in metabolism or genetic information processing categories, which will be useful for future characterization of the physiology, biochemistry, and functional genomics of *Uraria*.

Unigene-derived SSR markers have been widely used in studies of genetic diversity and population genetics, especially for phylogenetically related species [56–58]. In this study, polymorphic SSR markers of *U. lagopodioides* were developed using NGS technology. A total of 17,740 potential SSRs were identified from the set of 66,026 unigenes, with 26.9% of unigenes containing an SSR and an average distribution density of one SSR per 2.94 kb. The number and distribution density of SSRs in *U. lagopodioides* were significantly higher than those in *Argyranthemum broussonetii* (2.3% and 27 kb, respectively) [59], *Opisthopappus* (7.78% and 10.30 kb) [60], and *Arachis hypogaea* (17.7% and 3.30 kb) [61]. The differences in SSR abundance and frequency among different species may be partially attributed to the size of the unigene assembly dataset, SSR search criteria, sequence redundancy, database mining tools, and actual differences between species [62–64].

Among the identified SSRs, mono-nucleotide repeats are the most frequently observed, followed by tri-nucleotide and di-nucleotide repeats. For mono-nucleotide motifs, the proportion of the A/T motif (59.95%) was significantly higher than that of G/C (0.76%), which was consistent with most previous studies of other plants [25, 65–67]. The most abundant di-nucleotide motif was AG/TC (5.93%), followed by AT/TA (5.08%). The number of AT-containing repeats was significantly higher than that of GC-containing repeats, which suggests that these sequences are relatively unstable and prone to base substitution and gene mutation [68, 69]. The results of this study showed that the number of simple repeats of *U. lagopodioides* was negatively correlated with the size of SSR bases. Mono-nucleotide, di-nucleotide and tri-nucleotide repeats accounted for the majority of SSR loci ( 98.04%), while tetra-nucleotide, penta-nucleotide and hexa-nucleotide repeat unit combinations accounted for only 1.96%. The existence of a large number of short-repeat SSR loci may be due to the high mutation frequency and high rate of evolution of the genome itself. There are significant differences in the distribution of SSRs in different functional regions of the genome. SSRs located in the CDS region can affect gene activation and protein expression, while those located in the non-coding region and UTR region may affect gene regulation and translation.

Most previous studies of *Uraria* focused on their medicinal value [4, 6, 7], while studies on the taxonomy and evolution of *Uraria* were limited. DNA fragments involved in the previous phylogenetic studies were relatively conserved, limiting their value for analyses within *Uraria* [18, 19]. Therefore, unigene-derived SSR markers developed in the present study will be invaluable for further population genetic studies of *Uraria* species.

Using cluster analysis, 17 samples of seven *Uraria* species were clustered into two monophyletic clades, with samples from each species forming monophyletic clusters. *Uraria oblonga*, *U. lacei*, and *U. sinensis* were clustered in clade I. *U. lagopodioides*, *U. rufescens, U. crinita*, and *U. picta* were clustered in clade II. Interspecific relationships revealed by the cluster analysis based on the 19 unigene-derived SSRs were consistent with the inflorescence type of *Uraria* species. The inflorescence type of species in clade I is panicles, while that of species nested within clade II is racemes. The results of this study demonstrated that phylogenetic analysis based on unigene-derived SSRs can provide valuable evidence for the taxonomy and evolution of *Uraria*.

## Conclusions

In this study, we assembled and annotated a large number of unigenes of *U. lagopodioides* using RNA-Seq technology and also characterized and evaluated a number of unigene-derived SSR markers derived from the transcriptome of *U. lagopodioides*. A total of 54 unigene-derived SSRs were verified to be of cross-species transferability in *Uraria*, 19 of which displayed polymorphisms useful for phylogenetic studies. These results will provide a theoretical basis for further functional genomics, population genetics, and phylogenetic analyses of *Uraria* and relatives.

## Methods

### Plant materials and RNA / DNA extraction

The *U. lagopodioides* plant materials for RNA isolation and transcriptome sequencing were collected from Yuanjiang County, Yunnan Province in June 2018. Fresh leaf tissues were cleaned and immediately preserved in liquid nitrogen until RNA extraction. Total RNA was isolated using TRIzol Reagent (Invitrogen, CA, USA), and RNA purity was checked using the NanoPhotometer spectrophotometer (IMPLEN, CA, USA). RNA integrity was assessed using the RNA Nano 6000 Assay Kit on the Agilent Bioanalyzer 2100 system (Agilent Technologies, CA, USA) by Novogene (Beijing, China). The transcriptome of *Uraria lagopodioides* was sequenced using the Illumina HiSeq 2500 platform by Novogene (Beijing, China).

For identifying polymorphisms and testing the cross-species transferability of the developed unigene-derived SSR markers, 17 individuals representing seven *Uraria* species were sampled. Voucher information is provided in Table 3. The materials were identified by Dr. Xueli Zhao according to *Flora of China* [1] and deposited at the Herbarium of Southwest Forestry University (SWFU).Total genomic DNA was extracted from silica-gel-dried leaves with the TIANGEN plant genomic DNA extraction kit (TIANGEN Biotech, Beijing, China) following the manufacturer’s protocol.

**Table 3:** Voucher information of the materials used in this study

Species	Voucher	Locality
*U. lagopodioides* 1	WL (SWFU)	Qingyuan, Guangdong
*U. lagopodioides* 2	ZXL592 (SWFU)	Baise, Gunagxi
*U. lagopodioides* 3	ZXL441 (SWFU)	Chongzuo, Guangxi
*U. lagopodioides* 4	ZXL381-2 (SWFU)	Yvxi, Yunnan
*U. oblonga* 1	CB2019121901 (SWFU)	Changjiang, Hainan
*U. oblonga* 2	ZHL2019 (SWFU)	Dali, Yunnan
*U. oblonga* 3	ZXX20211003 (SWFU)	Maguan, Yunnan
*U. lacei*	WL201809 (SWFU)	Qingyuan, Guangdong
*U. picta* 1	ZXL663-3 (SWFU)	Chuxiong, Yunnan
*U. picta* 2	0911–1 (SWFU)	Chongzuo, Guangxi
*U. rufescens*	YLZB4792 (SWFU)	Mengla, Yunnan
*U. sinensis* 1	ZZM1143 (SWFU)	Leibo, Sichuan
*U. sinensis* 2	ZZM1045-2 (SWFU)	Gongshan, Yunnan
*U. sinensis* 3	ZZM1177-11 (SWFU)	Luding, Sichuan
*U. crinita* 1	ZXL558-1 (SWFU)	Huizhou, Guangdong
*U. crinita* 2	YLZB4783 (SWFU)	Mengla, Yunnan
*U. crinita* 3	DTT201804 (SWFU)	Wenchang, Hainan

### RNA-Seq library construction, sequencing, and transcriptome assembly

A total amount of 3 µg RNA per sample was used as input material for the RNA-Seq sample preparations. Sequencing libraries were generated using NEBNext Ultra RNA Library Prep Kit for Illumina (NEB, USA). mRNA was purified from total RNA using poly-T oligo-attached magnetic beads. Fragmentation buffer was added to mRNA samples, and these were then randomly sheared into 150–200 bp fragments. The library preparations were sequenced on an Illumina HiSeq 2500 platform by Novogene (Beijing, China). Transcriptome assembly was performed using Trinity [70]. The RNA-seq data have been submitted to the NCBI Sequence Read Archive (SRR21474487, https://www.ncbi.nlm.nih.gov/sra/SRR21474487). Gene function annotations using multiple databases were performed to obtain comprehensive gene function information. Diamond v0.8.22 (http://www.ncbi.nlm.nih.gov/COG/) was used to annotate gene functions via the KOG database with the parameter e-value = 1e-3. The KEGG (http://www.genome.jp/kegg/) Automatic Annotation Server was used for functional annotation of metabolic pathways and gene products, with the parameter set to e-value = 1e-10 [71]. Protein annotation analysis for GO was performed using Blast2GO v2.5 (http://www.geneontology.org/) with the parameter e-value = 1e-6.

### Unigene-derived SSR detection, primer design, and marker validation

Potential unigene-derived SSRs were screened using the program MISA 1.0 [72]. The mono-, di-, tri-, tetra-, penta-, and hexa-nucleotides were designed with minimum repeat numbers of 10, 6, 5, 5, 5, and 5 for the SSRs, respectively. 150 SSR primers with no more than 4 consecutive repeat units and a length greater than 18 nucleotides were randomly selected. These SSR primers were synthesized by Sangon Biotech (Shanghai, China).

These 150 unigene-derived SSRs were then tested for proper PCR amplification. PCR reactions were carried out with a 25 μL reaction volume containing 0.5 ng genomic DNA template, 1 μL of each primer (100 μM), 12.5 μL of 2 × SanTaq PCR Mix (Sangon Biotech, Shanghai, China), and 10 μL of ddH_2_O. PCR amplification conditions were as follows: initial denaturation at 94℃ for 5 min, followed by 35 cycles of 94℃ for 30 s, 54℃ for 35 s, and 72℃ for 60 s, and a final extension of 10 min at 72℃. PCR products were visualized via electrophoresis in 1% agarose gels and 8% polyacrylamide gels (Additional file 3: Fig. S1), and SSRs that could be successfully amplified were selected for polymorphism assessment.

The capillary electrophoresis (CE) PCR amplification was performed in a 25 μL solution containing 20–50 ng DNA, 0.5 μL of each forward primer (10 μM) labeled with a fluorescent dye (FAM, HEX, and TAMRA), 0.5 μL of unlabeled reverse primer (10 μM), 0.5μL of 5 μM dNTP (mix), 2.5 μL 10 × Taq Buffer (with MgCl_2_), and finally ddH_2_O to 25 μL. Amplification was performed with initial denaturation of 95℃ for 5 min, followed by 10 cycles of 94℃ for 30 s, 60℃ (-0.5℃/cycle) for 30 s, and 72℃ for 30 s. This was followed by a further 30 cycles of 94℃ for 30 s, 55℃ for 30 s, and 72℃ for 30 s, and a final extension of 10 min at 72℃. The amplification results of the SSR primers were analyzed with GeneMapper software (Applied Biosystems).

### SSR primer data and population genetic analyses

For SSR data analysis, CE products were manually scored based on allele size. Data were scored as “0” if no band was present and “1” if it was present. UPMGA cluster analysis was conducted using the NTSYSpc program [73].

## Supplementary Information

**Additional file 1: Table S1. Summary of SSRs identified from the tanscriptome of *U. lagopodioides.***

**Additional file 2: Table S2. Details of 54 transferable SSR markers.**

**Additional file 3: Fig. S1. Representative polyacrylamide gel profiles.**

## Data Availability

The Illumina NGS reads generated in this study have been submitted to the BioProject database of the National Center for Biotechnology Information (SRR21474487).

## Abbreviations

GO: Gene Ontology
KEGG: Kyoto Encyclopedia of Genes and Genomes
KOG: EuKaryotic Ortholog Groups
Pfam: Protein family
UPGMA: Unweighted Pair-Groups Method with Arithmetic Averages
Ta: Annealing temperature
